# Biomimetic Approaches in Clinical Endodontics

**DOI:** 10.3390/biomimetics7040229

**Published:** 2022-12-06

**Authors:** Naresh Kumar, Nazrah Maher, Faiza Amin, Hani Ghabbani, Muhammad Sohail Zafar, Francisco Javier Rodríguez-Lozano, Ricardo E. Oñate-Sánchez

**Affiliations:** 1 Department of Science of Dental Materials, Dr. Ishrat Ul Ebad Khan Institute of Oral Health Sciences, Dow University of Health Sciences, Karachi 74200, Pakistan; 2 Department of Science of Dental Materials, Dow Dental College, Dow University of Health Sciences, Karachi 74200, Pakistan; 3 Department of Restorative Dentistry, College of Dentistry, Taibah University, Al Madinah, Al Munawwarah 41311, Saudi Arabia; 4 Department of Dental Materials, Islamic International Dental College, Riphah International University, Islamabad 44000, Pakistan; 5 Department of Special Care in Dentistry, Hospital Morales Meseguer, IMIB-Arrixaca, University of Murcia, 30008 Murcia, Spain

**Keywords:** endodontia, regenerative endodontics, revascularization, revitalization

## Abstract

In the last few decades, biomimetic concepts have been widely adopted in various biomedical fields, including clinical dentistry. Endodontics is an important sub-branch of dentistry which deals with the different conditions of pulp to prevent tooth loss. Traditionally, common procedures, namely pulp capping, root canal treatment, apexification, and apexigonesis, have been considered for the treatment of different pulp conditions using selected materials. However, clinically to regenerate dental pulp, tissue engineering has been advocated as a feasible approach. Currently, new trends are emerging in terms of regenerative endodontics which have led to the replacement of diseased and non-vital teeth into the functional and healthy dentine-pulp complex. Root- canal therapy is the standard management option when dental pulp is damaged irreversibly. This treatment modality involves soft-tissue removal and then filling that gap through the obturation technique with a synthetic material. The formation of tubular dentine and pulp-like tissue formation occurs when stem cells are transplanted into the root canal with an appropriate scaffold material. To sum up tissue engineering approach includes three components: (1) scaffold, (2) differentiation, growth, and factors, and (3) the recruitment of stem cells within the pulp or from the periapical region. The aim of this paper is to thoroughly review and discuss various pulp-regenerative approaches and materials used in regenerative endodontics which may highlight the current trends and future research prospects in this particular area.

## 1. Introduction

The true concept of “biomimicry or biomimetics” is to develop manmade design while taking inspiration from nature [1]. Biomimicry is a Greek word (bios, meaning life, and mimesis, meaning to imitate), envisioned as a completely or partly induced biological phenomenon [2]. In the medical, dental, biotechnological, and pharmaceutical fields, the failure of conventional materials is due to the lack of the ability of these materials to follow a cellular pathway to fit in with biological systems [3].

In the 1950s, Otto Schmitt a biomedical engineer introduced the term “biomimetic” [4,5]. It is the Greek word “bio” meaning life, and “mimetic” is related to simulating or mirroring nature. The objective besides biomimetics was to produce biological materials and procedures that mimic nature [4,6]. Accumulation of inorganic ions with organic protein molecules is the basic concept of novel biomimetic approaches [7,8]. Therefore, the biomimetics approaches have involved the multi-translational areas of bioengineering, biology, chemistry, and materials sciences. Moreover, in the fabrication of various biomimetic materials, nanotechnology plays a major role [5,7] Clinically, biomimetics refers to mimicking the physiognomies of a natural tooth repair of affected dentition through biomimetic procedures and materials [7,9]. For example, to improve the osseointegration of dental implants, biomimetic-dental-implant coatings of calcium phosphate (CaP) and hydroxyapatite (HA) have been investigated and implemented [10,11]. Similarly, the biomimetic process is applied in adhesive-restorative materials that demonstrated esthetics mimicking natural teeth and tooth morphology. During the last decades, the restorative approach has steadily evolved, progressing from mechanical retention to advanced adhesion. Composite-resin materials and adhesive dentistry have become valuable tools on this context. The principles of biomimetic dentistry impose introduction of advanced composite-restorative materials to clinical practice, which should align with the nature and integrity of the tooth tissues [12,13]. Regeneration of oral tissues demonstrated promising results in tissue-engineering approaches [14,15,16]. Moreover, various endodontics procedures, including formation of dentin barrier by pulp-capping, root formation during apexogenesis or apexification, apical healing by root-end fillings, and pulp regeneration by cell-homing strategies [17,18], involve biomimetic approaches in endodontology.

Biomimetic dentistry is the art and science of restoring or repairing damaged teeth with various approaches that mimic natural dentition in terms of aesthetics and function. These approaches involve minimal invasive-dental management by the use of bioinspired materials to achieve remineralization [5]. Regenerative endodontics and tissue engineering are emerging and have the potential to repair damaged or partially developed teeth with normal pulp-dentin tissue [19]. This concept works by offering a natural extracellular matrix (ECM) simulating environment, signaling molecules, stem cells, and scaffolds. Consequently, the absence of pathology, pain, and the formation of root dentine is well-evident which indicates clinical success [20]. Contemporary endodontic regeneration involves a revascularization process in which the root-canal system is disinfected using the intracanal medicaments and a blood clot is formed by stimulating the tissues of the root apex. The presence of blood clots mimics a natural scaffold inside the root canal that facilitates the proliferation and differentiation of the pulp-dentin stem cells [20,21]. Moreover, the current concept of cell-homing supports the recruitment of pulp-apex tissue by endogenous-mesenchymal-stem cells [22,23]. In addition, several macromolecules are investigated to recruit endogenous-pulp cells by different approaches, including chemo-attractants, platelet-rich plasma, and ECM molecules [24]. Ideally, dental biomimetic materials should mimic the properties and functions of different parts of the tooth [25], sharing a common goal to recreate biological tissues and emphasizing using materials that simulate the biological effects of oral structures [26]. Commonly used biomaterials for dental-pulp-tissue engineering are collagen or poly(lactic) acid and hydrogel scaffolds. It is difficult to administer collagen into narrow pulp space. Moreover, to engineer dental pulp, various bioactive biomaterials, including synthetic and natural hydrogels, have been investigated for suitability [27], which are discussed in the following sections.

The ultimate outcome of regenerative endodontics is enhanced patient management which could be done by various strategies that translate the biological aspects of the regeneration of pulp into the clinical aspects. These clinical protocols varied from relating the natural ability of the pulp to heal to regenerating the affected pulp-dentin complex or achieving revascularization of the empty-root canal [20]. Therefore, the aim of this paper is to thoroughly review and discuss various biomimetic approaches for pulp regeneration and endodontic applications. In addition, current trends and future research prospects for translating biomimetic approaches to clinical endodontic applications are elaborated. 

## 2. Development of Regenerative Endodontic Procedures (REP)

In 1961 Nygaard-Østby explored, for the first time, the concept of treatment of necrotic pulp by regenerative endodontics [28]. The definition of regenerative endodontic procedures has been given by Murray and Gracia as “events based on biological design to substitute missing, diseased, underdeveloped or damaged components of the tooth structures including root and dentine structures to restore physiological functions of pulp dentine complex” [29,30]. The complete assembly involved in the regenerative endodontics procedures are stem cells, signalling molecules, and scaffolds harvested on the extracellular matrix (ECM) [20,21,31,32]. The essential goal in REP is to promote pulp-tissue regeneration, development of roots, and proliferation of the progenitor-stem cells from the bone/tooth region [33]. In the apical papilla, these osteo/odonto-progenitor-stem cells prevent the infection and necrosis of the root that is caused due to the proximity of the periodontal-blood supply [34]. In addition, REP may influence angiogenesis, cell survival, differentiation migration, and proliferation. Using mesenchymal-stem-cells-markers, regenerative endodontics procedures have been shown to have diverse potentials [35,36]. During the differentiation of endothelial-progenitor cells and the revascularization process, the immunostaining technique was used to identify the abundance of CD31/collagen-IV and vascular endothelial growth factor (VEGF), R2/Collagen-IV (10). Despite of lack of clinical trials of regenerative endodontics procedures in the literature, this modality of treatment is appreciated by clinicians globally. 

In dental-pulp regeneration, the necessary cells can be delivered either by cell transplantation or by cell homing [37,38]. A study conducted by Torabinejad et al. [39] found that, for successful pulp regeneration after the revascularization procedure, the presence of a 1–4 mm uninflamed tissue was beneficial. The study was conducted on immature- animal teeth [39]. Complete regeneration of pulp tissue with capillaries and neuronal cells have been found in the regeneration of canine pulp within 14 days in 2009. Iohara et al. [40] transplanted scaffolds loaded with collagen fibers (type I and III) and dental-pulp- stem cells. Moreover, after transplantation of the scaffold alone, there was “no engraftment at the pulpotomy site” that has been found [40]. Souron et al. [41] used rats’ molars in their study. They transplanted rats’ pulp cells in a scaffold comprised of type-I rat collagen. After one month of implantation, the living and mitotically-active fibroblasts, new vessels, and nerve fibers were observed where the pulp was seeded with cells, whereas a lack of cell colonization was found where the pulp was seeded with lysed cells [41].

In another study conducted by Jia et al. [42], simvastatin was injected, which is an inhibitor of the competitive 3-hydroxy-3-methylglutaryl coenzyme-A reductase. The scaffold used in their study was a gelatin sponge together with dental-pulpal-stem cells on extirpated pulps [42]. Simvastatin boosted the mineralization process and regeneration of the pulp and dentin after 10 weeks of implantation [42]. A combination of poly(l-lactic acid)/Matrigel scaffold with bone-marrow-mesenchymal-stem cells were placed on the extirpated pulp in a study conducted by Ito et al. [43] on immunosuppressed rats. A total of 15% EDTA and 1.5% sodium hypochlorite were used for rinsing the pulp chamber. After 14 days of implantation, complete pulp regeneration along with nestin-expressing odontoblast-like cells beneath the dentin was demonstrated. Nestin is a type VI intermediate-filament protein originally present in neural-stem cells [43]. To answer this controversy in today’s dentistry, American Association of Endodontics (AAE) considered regenerative endodontics as the most thrilling innovative expansion [35,44,45].

## 3. Revascularization or Revitalization

Teeth with apical periodontitis and immature root apex having periapical infection underwent the revascularization process in 1971 [46]. However, due to limitations in materials, instrumentation, and techniques, this attempt failed. However, with the constant innovations and developments of techniques, materials, and instruments now, several case reports [47,48] have used and incorporated this technique into everyday use with success. The process of revascularization technique is different from both apexification and apexogenesis [47,48]. Apexification is defined as ‘an apical barrier to avert the route of toxins and bacteria into periapical tissues from root canal” [5,49]. In most pulp-diseases scenario and apical periodontitis, calcium hydroxide is used. Due to its improving success rate, easy availability for the clinician and affordability for patients, it is considered one of the most important medicaments that have shown promising results [50,51]. Traditional apexification procedures were the only option for clinicians to treat pulpal necrosis of immature teeth before 2004 which presents a unique challenge to the dentist. Calcium-hydroxide dressing was considered the primary material to be used in these traditional apexification-treatment procedures. Apexification has proven to be highly foreseeable [5]. However, the disadvantage of this procedure is that over a period of months, it requires multiple appointments in addition to the higher incidence of cervical fracture [19]. ProRoot Mineral Trioxide Aggregate (MTA) is used in the artificial-apical- barrier technique to facilitate root-canal-obturation procedures [49]. 

When the pulp is inflamed with an incompletely developed tooth, apexogenesis is carried out [52]. Apexogenesis is a technique that discourses the inadequacies involved with capping the inflamed dental pulp. The objective of apexogenesis is the conservation of vital pulp tissue so that continuous development of roots with apical closure may occur. Calcium-hydroxide paste is placed as a wound dressing after removing most or all of the coronal pulp [53]. In recent years the treatment of necrotic-immature teeth has been changed due to the various pros and cons of apexification and artificial-barrier procedures. Revascularization is the terminology that is used to describe the treatment of immature-necrotic teeth which involves the proliferation of the tissues in the pulp space of the involved tooth [33]. When canal space is induced with bleeding, undifferentiated mesenchymal-stem cells accumulate significantly [54]. Thibodeau et al. and Wang et al. conducted various animal studies treating immature teeth with triple antibiotic paste and using the blood-clot technique in which the histopathological evaluations of the canal space have shown cementum and bone formation [55,56].

When the conventional apexification and apoxgenesis methods were compared with the regenerative endodontics in a retrospective study on immature-necrosed teeth, the survival rate of the revascularization-treated teeth was the highest [57]. However, other studies concluded that due to weak root structure in a significant number of cases the reliability, the success rate of these procedures was significantly poor [58]. Kahler et al. [59] concluded that the outcomes of 16 clinical cases [59] in which they compared conventional disinfection approaches with regenerative blood-clot induction. In this study, the authors found that continued root maturogenesis was reported in only two cases when observed radiographically. 

In the blood clots, Gomes-Filho et al. [60] incorporated bone-marrow aspirate, platelet-rich plasma, and artificial-hydrogel scaffold along with a basic fibroblast-growth factor. They took infected, fully developed, and over-instrumented teeth and found that the addition of PRP and bone-marrow aspirates into debrided root canals did not significantly improve tissue ingrowth. Moreover, they concluded that revascularization procedures in humans did not enhance the results by the addition of an artificial hydrogel scaffold combined with the basic fibroblast-growth factor [61]. A permanently immature tooth having apical periodontics and a sinus tract was treated with a revascularization technique in contrast with the apexification process; a positive enhancement of the results was demonstrated by Iwaya et al. [48,62]. In the case of necrotic pulp, an endodontic procedure was carried out to rejuvenate tooth vitality known as “revitalization”, while the replacement of lost or damaged pulp-dentin tissue complex is known as “regeneration [29]. However, the underlying mechanism for regeneration of the dentine-pulp complex is poorly understood. Instead, root-canal therapy may undergo a repair/healing process [63].

### 3.1. Advantages of the Revascularization Approach

1. Technically simple approach.
2. There is no need of using expensive biotechnology due to currently available instruments and medicament techniques.
3. There are almost negligible chances of immune rejection as this approach relies on the patient’s own blood.
4. Bacterial microleakage can be eliminated through the induction of stem cells into the root canal space, followed by the intra-canal barrier, inducing a blood clot.
5. The concerns of restoration retention need to be overcome.
6. When this approach is applied to immature teeth, it reinforces their root walls.
7. As the avulsed immature tooth has necrotic-pulp tissue along with an open apex, and short and intact roots; therefore, the newly formed tissue will easily reach the coronal-pulp horn because proliferation in a short distance is required. Therefore, the strategy behind the development of new tissue is to maintain the balance between the pulp-space infection and the proliferation of new tissue.
8. Additional growth of open-apex root takes place due to minimum instrumentation that will preserve viable pulp tissue.
9. The potential to regenerate more stem cells and the rapid capacity to heal the tissue in young patients needs to be recognised (Table 1).

### 3.2. Disadvantages of the Revascularization Approach

1. The origin of where the tissue has been regenerated from is yet to be known.
2. According to researchers, effective composition and concentration of cells are mandatory for tissue engineering. However, these cells are entombed in fibrin clots; therefore, researchers do not rely on blood-clot formation for tissue engineering function.
3. Treatment outcomes will be variable by the variations in the composition and concentration of the cells [64,65,66,67] (Table 1).

### 3.3. Prerequisites for Revascularization Approach (Figure 1)

Revascularization studies have established the following prerequisites:

- There should be open apices and necrotic pulp secondary to trauma.
- In addition, open apex should be less than 1.5 mm.
- The following agents can be incorporated to remove microorganisms from the canal.
  ○ Antibiotic paste
  ○ Calcium hydroxide [68]
  ○ Formocresol [69]
- The coronal seal should be effective.
- There should be a matrix or the growth of new tissues.
- When trying to induce bleeding, anaesthesia should be used without a vasoconstrictor [70].
- Canals should not be instrumented.
- Sodium hypochlorite should be used as the irrigant.
- There should be blood-clot formation (Table 1).

**Figure 1:**
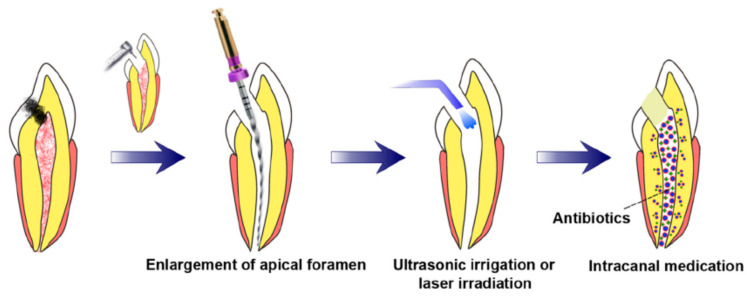
Requisite preconditions for pulp regeneration (root canal disinfection and enlargement of the apical foramen) [71].

## 4. Postnatal Stem Cell Therapy

Bone, buccal mucosa, fat, and skin are the common sources of postnatal-stem cells. After the apex is opened, the disinfected root-canal system is injected with postnatal-stem cells. This treatment is considered the simplest technique [72]. There are numerous benefits of this type of tissue-engineering technique. Postnatal-stem cells are rationally easy to harvest, and these cells can persuade the regeneration of the pulp. Moreover, these cells are easy to deliver by syringe. In addition, application of these stem-cell therapy is used in regenerative medicine since past many years, for example, bone-marrow replacement and endodontic applications [73]. However, low survival rates are one of the major disadvantages of this technique. Moreover, these cells can migrate into different locations of the body, which presents peculiar forms of mineralization [74]. For the development of dental tissues by the differentiation of stem cells, bioactive-signalling molecules, growth factors, and scaffolds are required [75]. Consequently, with only stem cells that exclude the growth factors or scaffolds, the chance of pulpal regeneration of new tissues is very low. In this approach, the chief identification of a postnatal-stem-cell source that must be able to differentiate into the diverse cell population can be obtained [74]. However, this technique is not approved yet. 

### 4.1. Pulp Implantation

In this procedure, after cleaning and shaping the root canal, the substituted pulp tissue is transplanted. Purified pulp-stem-cells line is among one the sources of the pulp tissue. This pulp tissue can also grow in the laboratory by cell biopsy. For this invitro technique, pulp tissues can be cultured by biodegradable-polymer nanofibers. Moreover, these tissues can be obtained from collagen I or fibronectin-extracellular-matrix proteins [76]. It has been found that further investigations are required for the proteins, such as vitronectin and laminin. However, it has been proved that for growing pulpal cells, collagens I and III are not fruitful [77]. In the root-canal system, the localization of postnatal-stem cells is a major advantage of pulp implantation. However, there are several disadvantages to this technique. It is a restriction of this technique that the apical portion of the root canal should be harvested with pulp cells. The reason behind this concept is that the sheets of the extracellular matrix are very thin, fragile, and they lack vascularity. Therefore, a scaffold that must have cellular proliferation is required for coronal delivery. If the cells are located 200 μm from a capillary- blood supply which is the maximum oxygen-diffusion distance, these cells are in danger of anoxia and necrosis. Further, in vivo investigation and controlled clinical trials are needed to explore the success rates and outcomes of functioning pulp tissue and concerns over immune responses, although this technique presents a low possibility of health risks to patients [78]. 

### 4.2. Scaffold Implantation

For vascularization and cell organization, pulp-stem cells must be systematized into a three-dimensional assembly. This objective can be achieved by seeding pulp-stem cells with a porous-polymer scaffold [79]. Distribution of therapeutic medicines to precise tissues can successfully be accomplished by these nano scaffolds [80]. Moreover, the biological and mechanical properties needed for proper functioning are also provided by these scaffolds [81]. In teeth that have pulp exposure, dentin chips have been introduced which accelerate dentin-bridge formation [82]. These dentin chips aid in the reservoir of growth factors and they offer a matrix for the attachment of pulp-stem cells [83,84]. In reaction to the dentine chip and the use of scaffolds, the regeneration of the pulp-dentin complex occurs. To provide structural support to the tooth it is not necessary to have a tissue-engineered pulp in the root-canal systems [85]. Polymer hydrogel, a soft three-dimensional injectable scaffold matrix, will be administrated by syringe in tissue-engineered pulp tissues [86]. They are easy to deliver into the root-canal systems and are non-invasive. Theoretically, this hydrogel provides a substrate for organized tissue structure as they are involved in cell proliferation and differentiation [87]. 

Recent advancements in these techniques overcome the problems associated with hydrogels. These issues comprised limited control over tissue development and formation [88]. However, further clinical trials and research are needed to explore these techniques as hydrogels are at an initial phase of exploration. Nowadays, researchers are focussing on photo-polymerizable-hydrogel development. The main advantage of these photo-polymerizable hydrogel is that the rigidity is enhanced by placing them into the tissue site [89]. 

### 4.3. Three-Dimensional Cell Printing

The three-dimensional cell printing technique is considered the final approach for the replacement of pulp tissues [90]. This approach can be used to position cells precisely [91]. This technique mimics the natural pulp-tissue structure. In tissue-engineering technique, to maintain and repair dentine, odontoblastoid cells should be positioned around the periphery of the pulp. Moreover, the fibroblasts support the vascular and nerve cells and should be positioned inside the pulp core. This technique required great expertise and careful orientation as during this procedure apical and coronal asymmetry is the prerequisite during the placement of the pulp tissue into the shaped and cleaned root-canal system. However, currently, this technique is not available clinically and there is a dearth of literature regarding the functionality of the three-dimensional cell-printing technique [92]. 

### 4.4. Gene Therapy

In regenerative endodontics, gene delivery has been discussed in a recent review [73]. To promote tissue mineralization, mineralizing genes would be delivered into the pulp tissues. However, Rutherford worked on this specific field of gene delivery into the pulp tissues, although there is a dearth of literature in this context [93]. He suggested further research to improve the possible gene therapy inside the pulp after he failed in his work when he transduced pulps of ferret animals with cDNA-transfected mouse BMP-7. Researchers used the electroporation method to insert mineralizing genes into the pulp space by culturing of pulpal-stem cells. Initially, the FDA approved the gene therapy research on terminally ill humans; however, after the development of numerous tumours in a nine-year-old boy, the FDA withdrew this decision in 2003. Gene therapy arising from the use of vector systems is posing serious health hazards in contrast to gene expressions [94,95]. According to the literature, tooth development can be enhanced with bone morphogenetic proteins (BMPs). Bone morphogenetic protein-2 (BMP2) is increased during the terminal differentiation of odontoblasts expression [96]. Dentin sialophosphoprotein (DSPP) has been produced by the implantation of human recombinant BMP2 on the dental papilla. The ultimate role of this DSPP is to produce the differentiation markers of odontoblasts as well as dentin-matrix proteins. An in vivo study on the amputated pulp, a large amount of reparative dentin is also induced by the BMP2 [96]. Clinically, cell-specific and safe-gene therapy is required to accurately control this gene therapy. 

### 4.5. Nitric Oxide

Among many wound healing and pathological processes, angiogenesis is considered an important process. The most potent and critical inducer of angiogenesis is the vascular endothelial growth factor (VEGF). A variety of stimuli take part in the regulation of gene expression of VEGF. The transcription factor is a key factor for hypoxia-mediated VEGF- gene upregulation, which is achieved by hypoxia-inducible factor 1 (HIF-1). Nitric oxide (NO) is a potent vasodilator. Nitric oxide (NO) can simply pervade natural membrane obstacles because it is lipophilic in nature. This VEGF regulates the amount of nitric oxide [97]. Hypoxia as well as nitric oxide upregulate the VEGF genes by enhancing HIF-1 activity. Moreover, dendrimers are released by nitric oxide which acts as antibacterial agents [98,99]. In one case, authors conducted a study in which they evaluated dendrimers with and without nitric oxide against Gram-positive and Gram-negative pathogenic bacteria. They used polypropylene imine (PPI) dendrimers that contained nitric oxide, which was compared with controlled PPI dendrimers that did not release nitric oxide. They found that >99.99% of bacterial strain was killed by dendrimers that contained nitric oxide. They further stated that there was minimal toxicity to mammalian fibroblasts with these nitric oxide-containing X dendrimers [98]. 

The most necessary clinical results of regenerative endodontics can be obtained by successful disinfection along with complete endodontic-tissue revascularization and regeneration. This was studied by Moon et al. [100]. However, there are many limitations of the contemporary regenerative endodontic procedure (REP). To improve the efficiency of regenerative endodontic procedure (REP), antibiotics and nitric oxide (NO) releasing biomimetic-nanomatrix gels have been developed very recently. The gel contains many functional groups as it is made up of peptide amphiphiles. This biomimetic- nanomatrix gel was mixed with antibiotics, ciprofloxacin (CF), and metronidazole (MN), and released nitric oxide. Multispecies-endodontic bacteria were used to evaluate the antibacterial effects by using bacterial-viability assays. Animal-model experiments were used to evaluate pulp-dentin regeneration. 

The concentration-dependent antibacterial effect was found in the antibiotics and NO-releasing biomimetic-nanomatrix gel. Moreover, nitric oxide without antibiotics also showed an antibacterial effect on endodontic species. Tooth revascularization has been promoted by antibiotics and NO-releasing biomimetic-nanomatrix gel by an in vivo analysis. To improve the current REP, an antibiotics and nitric oxide (NO) releasing biomimetic-nanomatrix gel was developed. For the regeneration-endodontics procedure, an optimum concentration of nitric oxide-releasing nanomatrix gel is recommended [100]. The positive or negative effects of nitric oxide may be attributed by changing the amount and concentration of nitric oxide. HIF-1 and VEGF activity was negatively affected by the release of nitric oxide. The activated endothelial nitric oxide synthase (EnoS) produces VEGF-mediated angiogenesis. Akt/PKB, Ca2+/calmodulin, and protein kinase C are the pathways by which eNOS get activated through VEGF. The NO-mediated VEGF expression as well as VEGF-mediated NO production by eNOS can be regulated by HIF-1 and heme oxygenase 1 (HO-1) activity. Angiogenesis in normal tissue can be regulated by the relations between NO and VEGF [97]. 

### 4.6. Platelet-Rich Plasma (PRP)

Special challenges are faced by clinicians for the treatment of an immature tooth with necrotic pulp and open apex. One of the strategies for its treatment is the traditional apexification procedure. This treatment process requires the formation of the apical barrier by multiple applications of calcium hydroxide. This apical barrier can also be formed by placing mineral trioxide aggregate (MTA) into the canal, which is followed by the conventional root-canal procedure [33]. Due to incomplete root formation with these procedures, the chances of root fracture are very common [19,33]. Platelet-rich plasma (PRP) has been suggested as probably the greatest platform for RET that will overcome all these problems [33,101]. Platelet-derived growth factor, transforming growth factor b, and insulin-like growth factor form an integral part of the PRP [5]. PRP can be utilized as a scaffold as it can form a three-dimensional fibrin matrix. It is easily prepared from the patient’s autologous whole blood [33,102,103,104]. Growth factors and cytokines are 4-fold higher in platelets than found in whole blood [105]. Mandibular-continuity defects, for the first time, were healed by the PRP and the placement of cancellous-bone grafts by the dental community [106]. Human dental pulp stem cells (DPSCs), when treated with PRP, resulted in an increase in the differentiation and proliferation of these cells [107].

In 2008, Hargreaves and colleagues [33] in regenerative endodontics encouraged the use of PRP, and for the first time in 2011, the PRP procedure was used for regenerative endodontics in a permanent, necrotic, immature, and nonvital tooth with an open apex. Infusion of PRP into the root canal up to the cementoenamel junction, followed by triple-antibiotics paste medication, was performed by Nakashima et al. [75]. They observed the closure of the apex and healing of periapical lesions after five and a half months. In addition, they also found encouraging results in electrical-pulp-testing-cold tests [25]. In a study conducted by Torabinejad M et al. [108], the authors injected PRP and observed that, after 14 months of the treatment, extirpated soft tissue was present that was evaluated through microscopy. Pulp-like connective tissue was also found in the microscopic section [108]. A study conducted on beagle dogs by Zhu et al. [109], in which authors infused PRP in endodontically prepared root canals, found the formation of cementum-like tissue and soft tissue [109]. In contrast, in a study conducted by Torabinejad et al., researchers found no significant difference when a root canal was treated with PRP in relation to soft-tissue formation [110]. In a 39-year-old female patient with necrotic pulp who has extensive periapical radiolucency and open apex after delivering PRP in a root canal, healing of the periapical lesion after 30 months of treatment was notified by the researchers [111]. 

**Figure 2:**
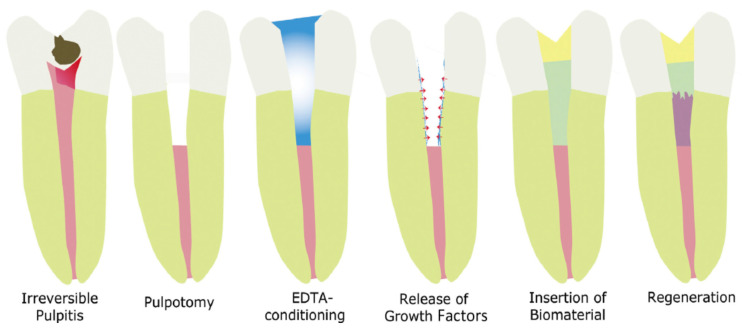
Clinical procedure for dental-pulp regeneration using a cell-free approach [119].

**Figure 3:**
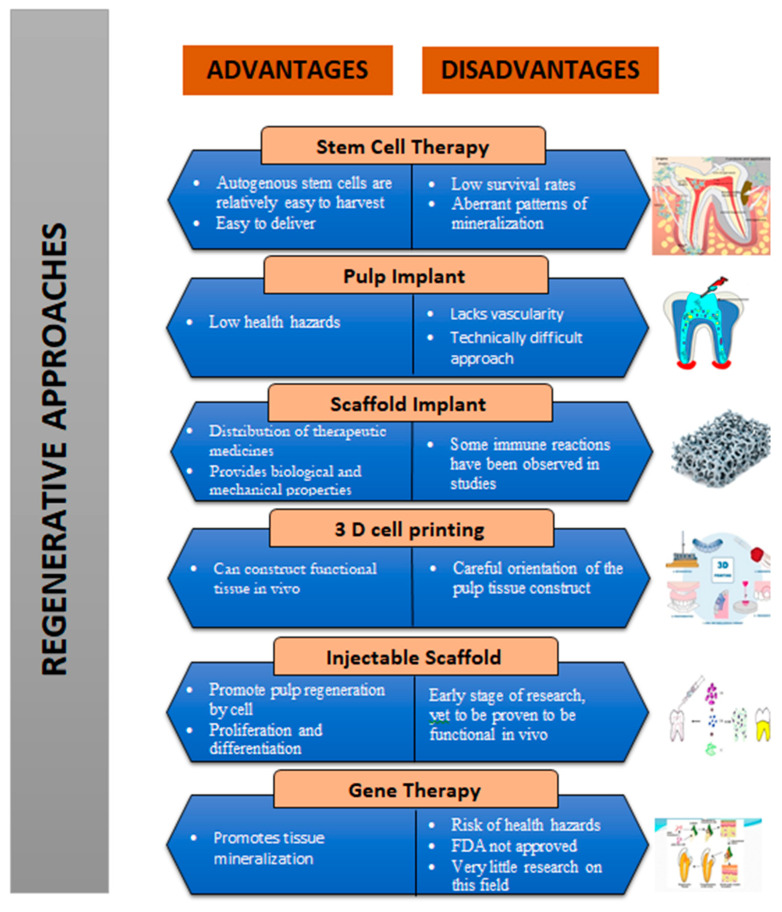
Summary of the advantages and disadvantages of the regenerative approaches in endodontics.

There are numerous advantages of PRP treatment. During the preparation of the PRP, erythrocytes that would be responsible for necrosis after clot formation was removed [33]. For cell migration, fibrin, fibronectin, and vitronectin are required which is obtained from the formation of PRP clots [104]. Moreover, in regenerative-endodontic procedures, the optimal level of MTA placement is mandatory which can be done by the collagen matrix present in the PRP [70,108]. Before clot formation, PRP does not release growth factor until it is activated. As soon as it is activated either endogenously or through the exogenous, such as by incorporation of calcium chloride or thrombin that acts as a clotting factor, PRP will start secreting growth factors that contribute to the repair and regeneration of the tissues [104,112]. Zhang et al. [113] concluded that histologically, no significant difference was observed between blood clots and PRP. PRP can be employed in clinical cases when little or no bleeding is observed from apical tissues. The source of stem cells, their interaction, and the role of inflammatory cells in the root canal need further exploration for the advancement of regenerative-endodontic treatment (Figure 2 and Figure 3). 

### 4.7. Cell Homing 

In tissue regeneration, the first concept of cell homing was presented in Lancet in 2010. The concept was based on the delivery of transforming growth factor-b3 (TGFb3) without cell transplantation. This approach was first used for the regeneration of the articular cartilage [114]. However, for dental-tissue regeneration, the idea of cell homing was introduced in 2010 [115]. During cell homing, root canal of the extracted human teeth was shaped and cleaned followed by the delivery of the growth factors, scaffold, and stem cells. Residual proteins in the root canal or dentinal tubules were deactivated in the first phase. This can be done by sterilization of extracted teeth in an autoclave. This was followed by the infusion of collagen gel into a shaped and cleaned root canal that might be with or without basic fibroblast growth factors (bFGFs), vascular endothelial growth factors (VEGFs), platelet-derived growth factors (PDGFs), nerve growth factors (NGFs), or bone morphogenetic proteins (BMPs). The animal model that was chosen by the authors was Sprague-Dawley rats in which both the experimental and control teeth were subcutaneously implanted for three to six weeks. The authors in this model observed the endodontically treated root for the dental pulp-like tissue having blood vessels. Enzyme-linked immunosorbent assay (ELISA) was performed on the soft tissues isolated from the root canal of both treated and control teeth. Various biomolecules, including dentin sialoprotein, NGF2, and von Willebrand factor, were quantified using the ELISA. Moreover, blood vessels, dentin-like tissue, and neural-like tissue are all found in human teeth after growth-factor delivery. These procedures do not involve ex-vivo cultivation or in-vivo transplantation [115]. 

The prime difference between the cell homing and cell transplantation approaches is that, in the latter case, for dentine/pulp regeneration, the isolated cells (stem/progenitor) from the host are transplanted into the root canal of the host. Dental pulp-like cells have been differentiated in the cell-homing approach when growth factors are recruited into the root-canal system. Cell-homing-technique-dental-organ regeneration presents a harmonizing and/or balancing approach to cell-transplantation technique and, at the same time, this strategy has shown auspicious results in animal models [23,115,116]. Hematopoietic-stem cells were militarized and transferred to different tissues or organs using active navigation in the cell-homing approach. The ultimate outcome of this process is pulp-dentin re-cellularization and revascularization. Numerous growth factors along with cell homing will result in pulp-dentin regeneration. Tissue revascularization and regeneration-cell homing consist of two distinctive cellular processes. They are differentiation and recruitment [117,118].

Migration of the cells to the defective site is referred to as recruitment. However, the presence of a mesenchymal stem/progenitor having the ability to differentiate into cells that form pulp and dentine is mandatory [117,118]. When stem/progenitor cells are transformed into mature cells, this process is known as differentiation. During pulp and dentine regeneration, odontoblasts and pulp fibroblasts are formed by the differentiation of the stem/progenitor cells. These processes need to persuade the budding of endothelial cells and neural-fibril cells for angiogenesis. However, more literature is needed to establish the fact that endothelial cells are formed directly by the differentiation of the dental pulp stem/progenitor [117,118]. In a study conducted by Kim et al. [115], the researchers implanted endontically treated human teeth into mouse dorsum. Fibroblast growth factor and/or vascular endothelial growth factor (bFGF and/or VEGF) were delivered [115]. After evaluation, they found that dentinal walls of the root canal were integrated with recellularized and revascularized connective tissue. Moreover, it was found that when bFGF, bone morphogenetic protein-7 (BMP7), platelet-derived growth factor (PDGF), VEGF, and nerve growth factor (NGF) were delivered in combination, it resulted in vascularized and cellularized tissues. These results were obtained with some endodontically treated teeth in which dentine formation and VEGF antibody were found in the dentinal wall of the root canal. 

Extracellular matrix with disconnected cells is found on neo-pulp tissues. This new pulp tissue appears to be dense and contains erythrocyte-filled blood vessels having endothelial-like cell lining. When bFGF, VEGF, PDGF, NGF, and BMP7 are delivered and the entire root canal is filled with dental pulp-like tissues found in microscopic images. Von Willebrand factor, dentin sialoprotein, and NGF are expanded in the ELISA after the combinatory delivery of bFGF, VEGF, or PDGF, with basal NGF and BMP7.

## 5. Biomimetic Materials in Endodontics

### 5.1. Biointeractive Materials

Bio-interactive material is the one that elicits a specific response by releasing biologically relevant ions [120].

#### 5.1.1. Calcium Hydroxide

Calcium hydroxide Ca(OH)_2_ was first introduced in 1920 by Herman as a pulp-capping agent. Since then, it has been used extensively in the field of endodontics. It is a solid compound with a high pH (>11) and can dissociate into Ca and OH ions. These ions are responsible for the therapeutic properties on tissues and bacteria, enabling them to act as an antibacterial, anti-inflammatory, and remineralizing agents [121]. Because of its alkaline pH, Ca(OH)_2_ possesses antibacterial properties. It lethally affects bacterial cells by damaging the cytoplasmic membrane, denaturing proteins, or damaging DNA [122]. It has a wide range of antimicrobial activity but has limited efficacy against *E. Faecalis* and *C. Albicans* [123]. Ca(OH)_2_ also inactivates endotoxin, which initiates inflammation and resorption [124,125,126]. In addition, the alkaline pH neutralizes acid from osteoclast, activates alkaline phosphatase, and the availability of Ca ion, which all play a vital role in the formation of hard tissue and in inhibiting resorptive activity [125,126]. However, the long-term placement of Ca(OH)_2_ may weaken the root dentine and even lead to cervical-root fracture [127].

Regarding regenerative endodontics, disinfecting the root canal with Ca(OH)_2_ promotes the proliferation of stem cells of the apical papilla (SCAP) [128,129] and increases the release of growth factors from dentine [130,131]. An in vitro study found that the Ca(OH)_2_ at a concentration of 1 mg/mL in the culture medium promotes the survival and proliferation of SCAPs [132]. Controlled-release calcium hydroxide-loaded microcapsules based on polylactic and ethylcellulose have been developed to improve their biological performance [132]. Such systems ensure the slow and sustained release of calcium and hydroxide over an extended period. The calcified barrier formed by the Ca (OH)_2_ is permeable and weak, and multiple soft-tissue inclusions create tunnel defects. The high dissolvability disintegrates material over time, leaving voids that can be the potential pathway for bacterial infiltration [133,134,135,136]. Highly alkaline pH also reduces the fracture resistance of dentine and negates its application for a prolonged time [137].

#### 5.1.2. Calcium Sulfate

Calcium sulfate, a natural mineral, has been widely used in orthopedics and dentistry to repair bony defects. It is biocompatible, bioresorbable, and osteoconductive. It undergoes complete and rapid resorption without eliciting any inflammatory-tissue response [138]. Generally, it is found in three distinct forms: CS dihydrate, CS hemihydrate, and CS anhydrite. In dentistry, hemihydrate form is used, which, after mixing with water, hardens in a slight exothermic reaction [139]. Calcium sulfate encourages the formation of bone through dissolution [140]. Rapid resorption results in a porous structure that serves as the scaffold for bony growth [141]. Dissolution causes the release of calcium ions which stimulates the osteoblast and inhibits the action of the osteoclast [142].

Chen et al. [143] used CS to repair large-bone defects in dogs’ tibiae, and the treated area showed new-bone formation without foreign-body reaction [143]. Peltier and Jones [144] also found the same results when they used it in patients to fill bony cavities formed by the removal of the unicameral-bone cyst [144]. Yoshikawa et al. [145] obtained favorable results when CS was used to treat osseous defects formed after apicectomy in beagle dogs [145]. Pecora et al. [146] also successfully used calcium sulfate as a bone graft in the surgical treatment of periradicular lesions [146]. In vivo studies have proved that calcium sulfate can induce new-bone formation when placed in bony defects. However, it lacks osteoinductivity, and the proteins present in blood and tissue fluids prolong its setting time. Rapid resorption sometimes hinders its use in large-bone defects [147], and thus, limiting its use in endodontics. To overcome these constraints, biphasic calcium sulfate and composites of calcium sulfate with other bio-materials are also available [148,149,150].

### 5.2. Bioactive Materials

“A bioactive material is one that elicits a specific biological response at the interface of the material which results in the formation of a bond between the tissues and the material” [151].

#### 5.2.1. Calcium Silicate Based-Cements

##### Mineral Trioxide Aggregate

Tora Binejad developed MTA in 1993 at Loma Linda University [152], which is composed of purified Portland cement (75%), Bismuth oxide (20%), and Gypsum (5%). Tricalcium silicate, dicalcium silicate, and tricalcium aluminate are the main components of Portland cement [153]. It can set in the presence of moisture, which is omnipresent in the oral cavity. MTA is mixed with water or saline, forming calcium-silicate-hydrate gel and calcium hydroxide. The pH of the set material is 12.5, which is comparable with that of calcium hydroxide [154,155]. It was primarily developed for root-end filling and perforations repair [156], but its immense clinical success has extended its application in various endodontic procedures [157,158].

The properties which favor its application in endodontic procedures are: high biocompatibility, bioactivity, excellent sealing ability, low solubility, and hydrophilicity [159]. MTA releases calcium ions in contact with human tissues and promotes osteoblast proliferation. It causes cytokine production from osteoblast, which favors the migration and differentiation of bone tissue-forming cells, indicating its remineralization potential [160]. MTA encourages reparative-dentine formation, which is rapid and thicker with good structural integrity [161], and a milder degree of pulpal inflammation while maintaining the integrity of the pulp [162,163]. An in vivo study found that the MTA-capped teeth resulted in more sialoprotein expression from dentine than Ca(OH)_2_ [164].

Hilton et al. [165] published a study about the clinical and radiographic difference between MTA and Ca(OH)_2_, and found that the MTA had a lower failure rate [165]. In addition, alkaline pH creates an antibacterial environment. MTA can activate cementoblasts for cementum formation and regeneration [166]. MTA is used as a coronal barrier in the final step of the regenerative endodontic procedure. Wattanapakkavong and Srisuwan [167] evaluated the effect of MTA on transforming growth factor beta 1 (TGF-b1) released from root-canal-dentine and human-apical-cell (APC) differentiation and mineralization after placing it as a coronal barrier in REPs. They found that it can cause an increase in the release of TGF-b1 and APC mineralization [167]. Gandolfi et al. and Torreira et al. [168,169] investigated the bone response after implantation of MTA in the bony cavity and found that it can induce bone regeneration and osteoinductive potential [168,169]. These studies support the possibility of expanding the clinical use of MTA as bone-repairing material. MTA also possesses negative characteristics, such as long setting time (2 h 45 min), sandy consistency (poor handling characteristics), poor dispersion, high porosity, low compressive strength, tooth staining, and high cost [170].

##### Biodentine

Biodentine was introduced in 2009 as a dentine-replacement material. It is formulated using MTA-based technology to improve the physical, setting, and handling properties while providing the same range of clinical applications of MTA [171]. It is a tricalcium-silicate-based-cement, having two additional components in the liquid: calcium chloride as a setting accelerator and hydrosoluble polymer as a water-reducing agent. It sets in around 12 min [172]. Set material contains Ca, OH, and silicate ions, responsible for their antibacterial and regenerative property. Micromechanical adhesion of biodentine crystals with underlying dentine provides favorable mechanical properties [173]. Wattanapakkavong and Srisuwan [167] demonstrated that BD has a better mineralizing potential than MTA as it can release a greater concentration of TGF-b1 from dentine. [167]. Luo et al. [174] found that biodentine increases the proliferation, migration, and adhesion of human-dental-pulp-stem cells, promoting remineralization [174]. Chang et al. [175] found that biodentine can induce odontoblastic differentiation of human-dental- pulp-stem cells obtained from impacted third molars [175]. Prior investigations have also expressed that the BD provides more calcium ions than MTA [176,177]. 

Grech et al. [178] showed that biodentine has the highest compressive strength compared to other cements due to its low water/powder ratio [178]. Set material also has lower porosity due to low water content [179]. Biodentine possesses the lowest solubility [178], superior sealing ability [180], antimicrobial action [181], and suitable biocompatibility [182]. 

Biodentine was used in a randomized clinical trial (RCT) as a pulpotomy agent in 41 primary molars and reported 100% clinical and 94.9% radiographic success after 12 months [183]. Another RCT used biodentine on 25 primary molars and reported 95.2% clinical and 94.4% radiographic success after 18 months [184]. Biodentine can also be used as a barrier material in regenerative endodontics. Topçuoğlu and Topçuoğlu [185] reported clinical cases in which biodentine was successfully used as a barrier in the management of necrotic-immature-mandibular teeth [185]. It has overcome the limitation associated with MTA. However, due to the lack of long-term observational studies, it is difficult to deduce clearly which material out of MTA and biodentine is superior, but we may conclude that economic factors, ease of manipulation, and fast setting time fall in favor of biodentine.

##### Calcium Aluminate Cement

Calcium-aluminate cement was created at the Federal University of Sao Carlos, Brazil [186]. It is mainly composed of calcium aluminate and calcium dialuminate, which is responsible for its hydraulic-setting reaction [187]. Upon mixing with water, it forms calcium-aluminate hydrate and aluminium hydroxide. The further decomposition of calcium-aluminate hydrate releases Ca and OH ions at a slower rate, producing an alkaline medium and providing therapeutic properties [187].

Pandolfelli et al. and Jacobovitz et al. [170,171,172,173,174,175,176,177,178,179,180,181,182,183,184,185,186,187,188] showed that the calcium-aluminate cement presents adequate biological and antimicrobial properties [170,188]. Garcia et al. [189] assessed the mechanical properties of the cement and found that it possesses higher compressive strength, diametral tensile strength, and microhardness value than MTA [189]. Oliveira et al. [190] evaluated the physical, chemical, and mechanical properties of the cement by incorporating different additives i.e., dispersant, plasticizer, and radiopacifier. They found that by adding these components, it resulted in a cement that sets more rapidly, has better fluidity and handling characteristics, better mechanical strength, and lower porosity than MTA. Lithium bicarbonate also reduced the setting time to 10 min from 60 min [190].

Larissa et al. [191] investigated the effect of calcium aluminate and MTA cement on osteogenic cells and found that both supported osteogenic-cell adhesion, spreading, and proliferation, but CAC-exposed cultures showed significantly higher values [191]. Lucas et al. [192] evaluated and compared the repair of bone defects filled with calcium-aluminate cement, MTA, and calcium hydroxide. It was an in vivo study, and the result showed that the MTA and calcium-aluminate cement resulted in the complete repair of bone defects created in rat tibias [192]. 

##### Theracal

Theracal is a new light-cured resin-modified calcium silicate-based biomaterial containing 45% mineral (Portland cement), 10% radiopaque agent, 5% thickening agent, and 45% resin [193]. It was specially designed for direct/indirect pulp-capping procedures, combining the excellent biological properties of calcium silicates, superior handling characteristics, and setting properties of the resin [194,195].

It releases calcium ions that favor the formation of apatite layer and mineralized tissues. Ca releases are in the concentration range of exerting a potential stimulating effect on dental pulp and odontoblast [196,197,198]. It has the ability to alkalinize the surrounding environment to approximately ph 10-11 and has lower solubility than MTA and Dycal. It can be cured to a depth of 1.7 mm [194]. Command setting facilitates the placement of final restoration with no delay. These attributes are of great help in the pulp-capping procedure. 

Lee et al. [199] evaluated the pulpal responses to TheraCal in dog-partial-pulpotomy cases and found that it produced extensive pulpal inflammation [199]. Hebling et al. [200] also determined the cytotoxic effects of resin-based light-cured cements on pulp cells and reported that all were toxic to the cultured odontoblast cells [200]. Jeanneau et al. [201] showed the outcome of adding resin to calcium silicates by evaluating TheraCal and Biodentine’s relationship with the pulp and found that Theracal is toxic to pulp fibroblast, produces a higher inflammatory reaction, and has lower bioactivity than biodentine [201]. Bakhtiar et al. [202] compared the use of TheraCal, MTA, and Biodentine for partial pulpotomy of human third molars, and the results showed that the TheraCal resulted in pulp disorganization and discontinued dentinal bridge [202]. Jeanneau et al. and Bakhtiar et al., based on their study, recommended not to use it for direct pulp capping and pulpotomy cases, and found MTA and Biodentine to be the more reliable choices of material. However, TheraCal has also been reported successful in short-term studies. A two-year in vivo study showed that TheraCal had a success rate of 93.3% for direct pulp capping, compared to GIC and antibacterial adhesive systems [203]. An in-vitro study estimated the marginal adaptation, solubility, and biocompatibility of TheraCal, MTA, and Biodentine as furcation-repair biomaterial. This study showed that TheraCal was the least soluble but showed the highest inflammatory response and distribution of gaps present, and recommended not be used in furcation-perforation repair [204]. 

#### 5.2.2. Calcium Phosphate Based Cements

##### Hydroxyapatite

Hydroxyapatite can be used in bulk form or as a coating for many biomaterials [205,206,207]. Its biocompatibility, osteoconduction, and osseointegration characteristics are well known. Due to its positive features, this material has remained the material of choice in the fields of dentistry as well as medicine for a long time [208,209,210]. The composition of synthetic hydroxyapatite is identical to the calcified part of teeth and bone [211]; as a result, this material is frequently used for dental and medical applications [212]. Despite the favourable bioactive and osteoconductive properties [213], inferior mechanical strength and toughness of hydroxyapatite prevent its applications under greater masticatory load areas [214]. 

Hydroxyapatite has been used in various clinical and animal studies with great success for the management of perforations, pulp capping and periapical defects [215,216,217,218]. Jean et al. [215] observed a greater degree of mineralization with tricalcium phosphate-hydroxyapatite compared to calcium hydroxide. Calcium hydroxide-based intracanal medicaments are also considered in regenerative endodontics [219], but they are responsible for root fracture. The combination of calcium hydroxide and hydroxyapatite has been suggested for the regeneration and repair of hard dental tissues [12]. Moreover, Zn-substituted hydroxyapatite has revealed enhanced bioactivity which makes zinc beneficial for therapeutic applications in the regeneration of hard tissues [220].

##### Bioactive Glass

Since the introduction of bioactive glass (BG) by Larry L. Hench, it has gained wide acceptance in the fields of medicine and dentistry [221]. BG has a noncrystalline structure and it shows relatively better bioactivity, compared to the other types of bioceramics with a crystalline structure. The BG is predominantly consisted of CaO, SiO_2_, and Na_2_O, and has the ability to proliferate, differentiate, and mineralize the human-dental-pulp cells [222,223,224]. The BG has commonly used to repair periodontal and bony defects. After its placement into the defects, it leads to different biological reactions which ultimately cause the remodelling and transformation of the living matrix and replace the same with fresh osseous tissues [225,226].

BG-based root-canal sealer, namely GuttaFlow Bioseal (GFB), has been made available to clinicians by (Coltène/Whaledent AG, Altstätten, The Switzerland). It exhibits low porosity, biocompatibility, and dentin penetrability. The other sealer, namely Nishika Canal Sealer BG (CS-BG) that is based on BG, has also shown sealing ability, biocompatibility, and better chemo-physical properties. Both sealers are gaining recognition among endodontists for the management of various endodontic issues [227,228,229]. In an in-vitro study, BG particles revealed a greater concentration of mineralized nodules [229]. In another study, BG aided the creation of a dense dentin barrier comparable to MTA [230]. 

Root-canal treatment is considered when microorganisms enter the pulp. A dimensionally stable and strong root-filling material is essential for the achievement of a tight coronal seal and the prevention of bacterial contamination [231,232]. To fulfil these requirements, BGs have been combined with polymer-based root-filling materials, such as Resilon [233]. Bio-gutta has been marketed which is basically the combination of conventional gutta-percha (GP) and BG, and it does not require any sealers during obturation [234]. Bio-gutta has a high degree of biocompatibility [235] and it allows the development of calcium phosphate, which in turn precipitates on the surface of the material under wet conditions and provides a tight seal [236,237]. In an in-vitro study, BG was combined with up to 30 wt.% Polyisoprene (PI) and polycaprolactone (PCL) separately so as to aid the development of root-canal-obturating materials that have a greater sealing characteristic. In addition, researchers compared these experimental groups with GP and Resilon as control groups and observed improved sealing ability with the former [238]. 

#### 5.2.3. Mixture of Calcium Silicate and Phosphate Based-Cements

##### Bioaggregate

Bioaggregate is recently introduced as a calcium-silicate-based endodontic cement, claiming to present an improved performance than MTA [239]. It is developed using the science of nano-technology and consists of nanosized hydrophilic particles of tricalcium silicate, dicalcium silicate, and tantalum oxide as a radiopacifying agent [240]. It is an insoluble, radiopaque, and aluminum-free material [241], which takes approximately 4 h to set completely [14]. The biocompatibility and sealing ability of the material are comparable to MTA [242,243,244,245,246]. Its ability to promote cementogenesis [247], coupled with bioactive nature, promotes apatite formation at the material/dentine interface, forming an impermeable seal [246]. Tuloglu and Bayrak [248] evaluated the clinical success of MTA and Bioaggregate as an apical barrier material and concluded that Bioaggregate could be an alternative to MTA [248].

An in-vitro study showed that the Bioaggregate-induced differentiation of human-pulp cells into odontoblast like-cells and can stimulate dentine-bridge formation [249]. Zang et al. [241] also found the same result. It has the lowest compressive strength amongst other calcium-silicate cements, making it less applicable in a clinical situation where adequate strength is required, such as furcation repair [250,251,252]. Despite its several advantages, poor mechanical properties and long setting time limit its application, where it can replace MTA.

##### Endosequence Root Repair Material

Root-canal perforations are either mechanical or pathologic communications between the external tooth surface, the root-canal system and their etiological factors, including caries, resorption, or iatrogenic [253,254]. To prevent continuous exposure to a contaminating environment and the occurrence of inflammatory reactions in the adjacent tissues [253], a material with good sealing ability should be employed [255]. MTA was introduced by Torabinejad and is considered a good material for creating an effective seal between root canals and outer dental surfaces [256,257,258]. Recently, biodentine has been marketed to address the deficiencies of MTA which include its difficult manipulation and extended setting time [171]. A newer premixed bioceramic material, namely ‘EndoSequence root repair material’, has been investigated for the management of apical surgery, perforation repair, pulp capping, and apical plug [259]. 

Kakani et al. [260] compared the sealing quality of MTA, Biodentine, and EndoSequence cements in perforations, and observed minimum, intermediate, and maximum leakage of Biodentine, EndoSequence, and MTA, respectively. The authors suggested that both Biodentine and Endosequence may be used as replacements for MTA during the repair of perforations. In 2019, Banu and Swathi [261] compared the Solubility of Endosequence root repair and MTA and found no significant difference. Sharma et al., in 2021 [262], assessed the Root Repair Material (ERRM), Endocem MTA, and ProRoot MTA, and observed a statistically significant difference between dentine-barrier thicknesses of the three groups. ProRoot MTA group revealed superior dentine-barrier thickness as compared to the other two groups. Hirschberg et al. [263] observed lesser apical leakage in MTA-restored samples as compared to ERRM. In a study by Hansen et al. [264], a higher pH was observed in white MTA specimens, compared to specimens repaired with EndoSequence Root Repair Material, and the authors attributed such effect to the greater and constant discharge of hydroxyl ions from the MTA samples. However, discolouration was seen in MTA specimens and such effect was not evident in the EndoSequence-Root-Repair specimens.

#### 5.2.4. Sealer

##### Endosequence BC Sealer

Calcium-silicate cements are now widely considered for pulp or periapical regeneration owing to their biocompatibility, bioactivity, antimicrobial properties, and sealing capability [265,266]. The biomineralization and biocompatibility characteristics of calcium silicate-based materials rendered them appropriate for a variety of applications, namely direct pulp capping [267], retrograde filling, and perforation repair [268]. These materials assist in pulpal healing by promoting the proliferation of stem cells of the dental pulp and the resultant formation of a dentine bridge [269,270,271]. In addition, a biological seal of the apical-root canal is also probable with these types of cement [272]. Based on the promising clinical as the well biological performance of these materials, new relevant endodontic sealers are being introduced with a couple of suitable bioactive and sealing properties.

Endosequence BC Sealer (BCS, Brasseler USA, Savannah, GA, USA) is an injectable calcium silicate-based material and it was introduced as a root-canal filling and sealing material [273]. It possesses suitable physicochemical properties and hardens in the presence of moist conditions [274]. To make this sealer suitable for use in the warm-canal-obturating techniques, the composition of Endosequence BC Sealer has been altered into Endosequence BC Sealer HiFlow (Brasseler, Savannah, GA, USA). This new sealer exhibits a lower viscosity on heating and is relatively more radiopaque than its predecessor. Moreover, the results of both BCHiF and BCS are comparable in terms of cell migration, cell adhesion, cytocompatibility, and bioactivity [275].

The cytotoxicity and the influence of heating on the physicochemical properties of BCHiF and BCS Sealers were compared by Chen et al. [276]. Their results show that the cell viability considerably declined on Day 3 for the 1:4 diluted extracts from both materials. The setting time, microhardness, and solubility of both sealers were comparable at 37 °C and 100 °C. However, BCHiF exhibited significantly higher flow and radiopacity compared to BC Sealer at room temperature, and the chemical composition of both sealers was not affected by the heating. 

Giacomino et al. [277] reported superior osteogenic potential and biocompatibility of EndoSequence BC Sealer (Brasseler, Savannah, GA, USA) and ProRoot ES (Dentsply Tulsa Dental Specialties, Johnson City, TN, USA), compared to Roth (Roth International, Chicago, IL, USA) and AH Plus (Dentsply DeTrey Gmbh, Konstanz, Germany). The results of their study showed that both bioceramic sealers have outstanding biocompatibility even at high concentrations, in contrast to Roth and AH Plus. Notably, both bioceramic sealers significantly improved osteoblastic differentiation through better responses observed with Endo- The sequence BC Sealer. On contrary, osteoblastic differentiation and function were significantly reduced with Roth or AH Plus sealers. Bukhari et al. in 2019 [266] compared the antibacterial performance of bioceramic and AH-Plus (Dentsply International Inc, York, PA, USA) sealers on 8-week-old *Enterococcus faecalis* biofilms adhered to the surface of root canals. Their results show that the EndoSequence BC Sealer is significantly more effective against the *E. faecalis* as compared to the AH plus sealer.

#### 5.2.5. Gutta-Percha

##### Bioceramic Coated Gutta-Percha

Gutta-percha points are extensively used as root-canal filling material [278]. However, their cytotoxicity has not been comprehensively evaluated. Some studies provide satisfactory reports regarding biocompatibility of gutta-percha [279] whereas others highlight a delayed healing and persistent periapical radiolucency owing to extruded gutta-percha [280]. To deal with the biocompatibility issue, alterations have been made in the composition of obturation points. EndoSequence BC points (Brasseler USA, Savannah, GA, USA) (BC) are bioactive substances coated on gutta-percha cones with bioceramic nanoparticles [281]. The bioceramic particles present in the EndoSequence Sealer bind to the bioceramic nanoparticles in the BC and form a gap-free filling in accordance with the reports of the manufacturer. 

In an in-vitro study conducted by Meneses et al. in 2019, [282] examined the cytotoxic potential of conventional gutta-percha (CGP) points, CPoint polymer (CP), and gutta-percha points containing bioceramics (BC) on periodontal ligament (PDL) cells. The findings of the study highlight greater toxicity of CP as compared to CGP; however, BC revealed no cytotoxicity. In 2018, Al-Haddad et al. [283] compared the push-out bond strength of apatite calcium phosphate coated gutta-percha (HAGP) with various commercial coated gutta-percha root obturation points. The authors identified the highest mean bond strength in HAGP compared with other commercially available coated gutta-percha root obturation points. In conclusion, it was suggested that due to the promising results of HAGP, it can be considered a suitable candidate for endodontic filling along with bioceramic sealer.

### 5.3. Remineralizing Agents

Remineralization is defined as the process whereby calcium and phosphate ions are supplied from a source external to the tooth to promote ion deposition into crystal voids in demineralized enamel to produce net mineral gain [284].

#### 5.3.1. Enamel Matrix Derivative (Emdogain) Remineralizing Agent

Enamel matrix derivative (EMD) is extracted from the buds of porcine teeth which mainly comprise amelogenins (about 90%) and smaller quantities of tuftelin, ameloblastin, enamelin, and other nonamelogenin proteins [285]. Its common clinical uses are stimulation of regeneration of periodontal attachment, and some clinicians have also suggested its potential for periodontal regeneration [286,287,288,289]. Several case reports related to the field of endodontics using EMD for perforation repair or root-end resection along with guided tissue regeneration are well evident [290,291,292]. The potential of EMD in endodontic regeneration is not fully agreed upon; however, its important role in odontogenesis via up-regulation of Osterix and Runx2 transcription factors is well-documented [289]. In addition, EMD increases the expression of markers for odontoblast cells in human- dental-pulp cells which may aid pulp-tissue repair and regeneration [293]. An animal study compared the reparative activity of calcium hydroxide Ca [294] and EMD for pulpal exposures, and the findings of the study revealed that the quantity of newly formed hard tissue in the EMD-treated teeth was twice that of the Ca(OH)_2_-treated teeth [295]. According to various in-vitro studies, it appears that EMD is capable of upregulating dentin matrix protein-1, dentin sialophosphoprotein, and osteopontin RNA in human-dentin- pulp-stem cells. Moreover, EMD may also be a suitable candidate for the treatment of cracked teeth, injured or avulsed teeth, and apexification [296,297,298,299,300]. 

#### 5.3.2. Dentine Matrix Derivative/Demineralized Dentin Matrix

The organic matrix of dentin consistsof a total of 233 proteins which include various collagenous and non-collagenous proteins [299]. Demineralized dentin matrix (DDM) is mechanically better, nonimmunogenic, and biocompatible, and has the potential for osteoinduction and osteoconduction [301,302,303]. It also allows the differentiation of odontoblast-like cells [304]. DDM is considered a complex of type I collagen (COL-I) and a growth factor, and it has significant osteoinductive and osteoconductive biological effects [305]. Various bone injuries and bone defects have been treated using autologous and xenogenous DDM [306,307,308]; however, the influence of DDM on DPSCs is not well documented. 

Liu et al., in 2016 [301], investigated the effect of DDM on DPSCs and the findings of their study indicate that DDM has the potential to stimulate DPSC odontoblastic differentiation, which make it a suitable candidate for dentin regeneration. The identification of appropriate scaffolding materials to assist cell growth and tissue regeneration is an important aspect of tooth-tissue engineering. Treated dentin matrix (TDM) has been demonstrated to be a suitable scaffold for rat-dentin regeneration. However, due to variations in species, the applicability of a similar fabrication method to human TDM and human dentin regeneration may be questioned. To deal with this area, Li et al., in 2011 [309], explored the biological response to a human TDM (hTDM) using various biological characteristics, namely cell proliferation, cell migration, cell viability, and cytotoxicity. The results of their investigation indicate that DFC attachment, growth, viability, and cytotoxicity on the surface of hTDM revealed a remarkable improvement in contrast to calcium-phosphate controls. The authors concluded that hTDM could be indicated as an ideal biomaterial for human-dentin regeneration. Chen et al. [310] developed a novel TDM paste as the pulp-capping agent and compared it to calcium hydroxide. The TDM paste showed better biocompatibility, and thicker and denser dentin-bridge formation in contrast to calcium hydroxide. Consequently, TDM paste could be considered a promising pulp capping agent.

### 5.4. Miscellaneous

#### Calcium Phosphate Cements

Calcium phosphate-based biomaterials have gotten increased attention due to their excellent biocompatibility, non-cytotoxicity, and chemical composition similar to teeth and bone [311,312,313,314]. Bioactive nature coupled with osteoinductive potential makes it a suitable material for endodontic applications [315]. It can chemically adhere to bone and teeth [316]. Brown and Chow first introduced the calcium-phosphate cement, a self-hardening cement consisting of a mixture of calcium phosphate which, when mixed with water, hardened into a less soluble calcium phosphate [317]. It has been widely used in orthopaedics and dentistry to repair bone defects, owing to its osteoconductive potential [318]. In-vitro studies have proposed its use for furcation repair, root-apex sealing, root- canal filling, and root-surface desensitization [319,320]. Anjali et al. [321] evaluated the efficacy of calcium phosphate cement in a single-visit-apexification procedure, and a 100% clinical success rate was observed [321]. 

However, the poor handling properties, low initial mechanical strength, and long setting time (around 60 min) of the cement limit its use in direct pulp-capping procedures [322,323,324]. Kai-Chun et al. [325] introduced biphasic calcium phosphate/sulphate cement, which has improved handling and its mechanical properties, and has a shorter setting time, and hence, making it a potential candidate for VPT [325]. Unlike Calcium silicates, calcium phosphate does not possess any anti-bacterial action. The incorporation of additives, such as hinokitiol and calcium silicates, can improve its anti-bacterial properties, and thus, opening the door for further endodontic applications [326].

## 6. Challenges in Regenerative Endodontics

The best treatment option for immature teeth with apical periodontitis is the revascularization process, which is an effective management strategy but technically challenging process. Following recommendations are made for effective revascularization that was based on different clinical case studies [70].

- To induce bleeding, the clinicians should use anaesthesia without a vasoconstrictor.
- As discussed earlier, to place biomimetic-endodontic materials at the controlled and optimal level, the matrix of collagen could be helpful.
- Specifically, when treating anterior teeth and generally for all dentitions, patients/parents should be informed about the staining potential as this paste contains minocycline.
- Patients/parents should be informed about the multiple visits for proper case selection [70].

## 7. Clinical Outcomes of Regenerative Endodontics Procedure

The clinical success of regenerative endodontic procedures, as defined by The American Association of Endodontists, is evaluated by the following three outcomes [327].

- Primary goal: This is an essential goal. It consists of elimination of signs, symptoms, and bony healing.
- Secondary goal: This is a desirable goal. In this, there will be increased root length and root-wall thickness.
- Tertiary goal: Vitality testing is positive.

It has been demonstrated that the primary goal of regenerative endodontics is generally achievable with high probabilities (91–94%) of success [328,329]. The failures might be due to minimal filing and disinfection protocols [330]. In the majority of studies, the secondary goal of regenerative endodontics is related to the narcotic pulp of immature permanent teeth that demonstrates the thickening of the canal walls and/or continued root development. However, it has been shown that these findings are not always predictable after RET treatment. Although it has been believed that, to strengthen the fragile immature permanent teeth and restore the vitality of the damaged tissue, RET was capable of regenerating the pulp–dentine complex by improving the thickness of the canal walls and the canal space [310,328]. However, the concept that the thickening of the canal walls after RET significantly reinforce the immature permanent teeth is not established by studies. It is just a radiographic assumption. Nevertheless, in a study conducted by Zhou et al., the researchers elaborated that fracture resistance of thickened canal walls was increased in an animal study [331]. The mechanism involved is that cell migration occurs from the apical papilla and remaining residual pulp to the disinfected canal space. This will lead to the deposition of dentine on canal walls and root apex which increases the thickness of the canal wall and root length. It was suggested that cells from the remaining residual pulp [47,68] or cells from the apical papilla [332] might migrate to the disinfected canal space to deposit dentine on the canal walls and the root apex, and thus increasing the thickness of the canal walls and length of the root [68]. 

## 8. Future Perspective on the Regeneration of the Dental Pulp

To date, successful stem-cell therapy is exemplified only in a few studies. For examples, blood reconstitution [333], corneal regeneration [334], and skin regeneration [335]. In tissue regeneration, the cell-homing approach can be observed in normal-tissue wound healing. In this process, chemotactic factor and stromal cell-derived factor 1 (SFF-1) are released [336] when tissue is injured. These factors signal nearby perivascular-stem cells and distant mesenchymal-stem cells to the site of tissue damage [337]. Based on studies on humans and animal, cell-based and cell-homing approaches can be utilized for the regenearation of dental pulp in regenerative endodontics. 

## 9. Conclusions

It appears that in-vitro and animal experiments regarding biomimetic approaches in regenerative endodontics are widely performed, but they are still at the inception stage. The findings related to stem-cell therapy, pulp implant, scaffold implant, 3D-cell printing, and gene therapy are quite promising as positive features with regard to pulp regeneration and tissue mineralization have been observed. Despite the aforementioned advantages, future developments in pulp-dentin tissue regeneration are needed to demonstrate the functional tissue regeneration and the ultimate favorable clinical benefits. In addition, some bioactive materials seem to be favorable as they promote osteoconduction and osseointegration, and are capable to proliferate, differentiate, and mineralize the human-dental-pulp cells. However, physico-mechanical characteristics of some materials are not satisfactory and warrant further investigations. Enamel matrix and dentine matrix derivatives have also been researched and their role in the dentine regeneration is encouraging, but there is a lack of scientifically validated data. 

## Figures and Tables

**Table 1:** Summary of advantages, disadvantages, and prerequisites of the revascularization approach.

Advantages [64,65,66,67]	Disadvantages [64,65,66,67]	Prerequisites [30,68,69,70]
Technically simple. Technique can be used without expensive biotechnology. Chances of immune rejection and pathogen transmission are negligible. Microleakage negligible Concerns of restoration retention are negligible. In immature teeth, the walls of the rots are reinforced. New developed tissues can easily access the root canal system. Preserve vital tissue as minimum instrumentation Rapid capacity to heal the tissue in young patients More regenerative potential in young patients	It is still not known from which source the tissue can be regenerated. Blood-clot formation is not the factor on which tissue engineering relies. Treatment outcome will vary with changes in the concentration and composition of cells.	Necrotic pulp secondary to trauma, open apices, and permanent dentition. Tooth must have thin walls. Effective coronal seal Matrix for the growth of new tissues Canal should not be instrumented. Blood-clot formation Sodium hypochlorite as an irrigant Non-vital traumatized tooth If treatment procedures, such as apexogenesis, apexification, partial pulpotomy, or root canal obturation, are not suitable, then only this treatment approach will be used.

## Data Availability

Not applicable.
